# High incidence of virulence determinants, aminoglycoside and vancomycin resistance in enterococci isolated from hospitalized patients in Northwest Iran

**DOI:** 10.1186/s12879-019-4395-3

**Published:** 2019-08-27

**Authors:** Fakhri Haghi, Vahid Lohrasbi, Habib Zeighami

**Affiliations:** 1 Department of Microbiology, School of Medicine, Zanjan University of Medical Sciences, Zanjan, Iran; 2 Zanjan Pharmaceutical Biotechnology Research Center, Zanjan University of Medical Sciences, Zanjan, Iran; 3 Department of Microbiology, Iran University of Medical Sciences, Tehran, Iran

**Keywords:** Aminoglycoside resistance, Enterococci, Vancomycin resistance, Virulence factors

## Abstract

**Background:**

Multidrug resistant (MDR) enterococci are important nosocomial pathogens causing serious problem in hospitalized patients. The aim of present study was to investigate the frequency of high-level aminoglycoside-resistant and vancomycin-resistant enterococci (VRE) and virulence encoding genes in enterococci isolated from hospitalized patients.

**Methods:**

A total of 100 enterococci isolated from urine samples of hospitalized patients with symptomatic urinary tract infections were investigated for antimicrobial susceptibility, the frequency of aminoglycoside and vancomycin resistance genes (including *aac (6′)-Ie-aph (2“)-Ia, aph (3’)-IIIa, ant (4’)-Ia, aph (2”)-Ic, aph (2“)-Ib, aph (2”)-Id, ant (3″)-III, ant (6′)-Ia, vanA, vanB* and *vanC*) and virulence encoding genes (including *gelE, PAI, esp, ace, cyl, hyl* and *sprE*).

**Results:**

*Enterococcus faecalis* species was identified as predominant enterococci (69%), followed by “other” *Enterococcus* species (21%) and *E. faecium* (10%). Ninety three percent of isolates were resistant to one or more antimicrobial agents, with the most frequent resistance found against tetracycline (86%), ciprofloxacin (73%) and quinupristin-dalfopristin (53%). Gentamicin and streptomycin resistance were detected in 50 and 34% of isolates, respectively. The most prevalent aminoglycoside resistance genes were *ant (3″)-III* (78%) and *aph (3′)-IIIa* (67%). Vancomycin resistance was detected in 21% of isolates. All *E. faecium* isolates carried *vanA* gene, whereas, the *vanB* gene was not detected in *Enterococcus* species. The most frequent virulence gene was *ace* (88.6%), followed by *esp* (67.1%), *PAI* (45.5%) and *sprE* (41.7%).

**Conclusion:**

Our study revealed the high frequency of gentamycin resistance and VRE in *E. faecium* isolates, with a high prevalence and heterogeneity of virulence and resistance genes. Due to high frequency of MDR enterococci, it seems that the appropriate surveillance and control measures are essential to prevent the emergence and transmission of these isolates in hospitals.

## Background

Enterococci are the second most common causative agent of urinary tract infections (UTIs) in hospitalized patients [1, 2]. Antimicrobial resistance and survival ability in various hospital environments have made them as serious problem in nosocomial infections due to the limited therapeutic options [3, 4]. The inherent antibiotic resistance and dissemination of resistance genes through conjugative transposons and plasmids play an important role in development of multidrug resistant (MDR) enterococci [5].

Aminoglycosides alone are considered inactive in the treatment of enterococcal infections and are usually combined with inhibitors of cell wall synthesis such as vancomycin or ampicillin [6]. High-level aminoglycoside-resistant (HLAR) and vancomycin-resistant enterococci (VRE) have created serious problems for antibiotic therapy [6]. Vancomycin-resistant enterococci are more common in North America, Europe, and Asia. Eight genotypes (*vanA, vanB, vanC, vanD*, *vanE*, *vanG, vanM* and *vanL*) have been described, of which, *vanA* (Tn1546) genotype with acquired inducible resistance to vancomycin and teicoplanin and *vanB* (Tn1549/Tn5382) genotype with variable resistance to vancomycin and susceptibility to teicoplanin are the most common [7].

High level aminoglycoside resistance is due to acquisition of genes encoding the aminoglycoside modifying enzymes (AMEs) such as aminoglycoside phosphoryl transferase (APH), aminoglycoside acetyl transferase (AAC) and aminoglycoside nucleotidyl transferase (ANT) [8]. The high level gentamicin resistance (HLGR, MIC≥500 μg/ml) is commonly due to *aac (6′)-Ie-aph (2″)-Ia*, which is located on the Tn5281 transposon and encodes a bifunctional enzyme, AAC (6′)-APH (2″) [8]. Recently, aminoglycoside modifying genes *aph (2″)-Ib, aph (2″)-Ic,* and *aph (2″)-Id* were detected among enterococci [9]. These genes are associated with high levels gentamicin resistance. Moreover, high-level streptomycin resistance (HLSR, MIC ≥2000 μg/ml) is mediated by *aph (3′)-IIIa* and *ant (6′)-Ia* genes [9].

Enterococci possess virulence genes including *ace*, *PAI*, *asa1*, *sprE*, *cylA*, *efaA*, *esp*, *gelE* and *hyl* encoding collagen-binding protein, pathogenicity islands, aggregation substance, serine protease, cytolysin, endocarditis antigen, enterococcal surface protein, gelatinase and hyaluronidase, respectively [1]. The gelatinase is an extracellular metalloprotease that hydrolyzes collagen, gelatin, and small peptides [10]. The enterococcal cytolysin is a member of bacteriocin family which lyses bacterial and eukaryotic cells in response to quorum sensing signals [11]. The enterococcal surface protein seems to contribute in the colonization and persistence of enterococci in ascending infections of the urinary tract and biofilm formation. Hyaluronidase is an important factor in nasopharyngeal colonization and pneumonia [10]. Recent studies showed the association between the presence of virulence factors and promoting emergence of enterococcal infections in nosocomial settings [5]. However, our knowledge about the possible relationship between the presence of virulence factors and their role in the emergence and development of resistance among enterococci is still limited [2]. Previous studies revealed that antimicrobial resistance and virulence are two different aspects of bacterial cell fitness and increased antimicrobial resistance might not always be associated with increased virulence [12].

Regarding the emergence of MDR enterococci have become a serious problem in hospitalized patients, the present study aimed to investigate the frequency of HLAR and VRE strains, antibiotic susceptibility, the frequency of AME and Van genes (including *aac (6′)-Ie-aph (2“)-Ia, aph (3’)-IIIa, ant (4’)-Ia, aph (2”)-Ic, aph (2“)-Ib, aph (2”)-Id, ant (3″)-III, ant (6′)-Ia, vanA, vanB* and *vanC*) and virulence encoding genes (including *gelE, PAI, esp, ace, cyl, hyl* and *sprE*) in enterococci isolated from urine samples.

## Methods

### Bacterial isolation and identification

Between March 2016 and February 2017, 1 hundred enterococci were isolated from urine samples of hospitalized patients with symptomatic urinary tract infections (UTIs) at least 48 h after hospital admission from three major hospitals in Zanjan, Iran. Informed consent and ethical approval was obtained from management of the hospitals prior to the study. The symptomatic UTI criteria consisted of dysuria, suprapubic pain or tenderness, urgency and frequency of micturition. The exclusion criteria for patients were fever, nausea, vomiting and mixed infection. Catheter urine samples were also excluded from our study. Laboratory confirmed UTI was defined as pyuria (> 10 WBC/mm^3^ per high-power field) plus bacteriuria (≥10^5^ cfu/mL). Urine samples were cultured on blood agar (Merck, Germany) and incubated under aerobic conditions at 37 °C for 24 h. Identification of isolates to the genus level was performed using Gram staining and biochemical tests. Species-level identification was performed by PCR targeting the *ddl* genes encoding D-alanine–D-alanine ligases specific for *E. faecalis* (*ddl*_*E.faecalis*_) and *E. faecium* (*ddl*_*E.faecium*_) (The primers are shown in Table 1). Verified enterococci were preserved at − 70 °C for further analysis. All Microbiological and molecular tests were performed in department of Microbiology, Zanjan University of Medical Sciences, Zanjan, Iran.

**Table 1:** Primers sequence and annealing tempratures used in this study

Target	Primer sequence (5′ → 3′)	Amplicon size (bp)	Annealing tempreture	Ref.
*ddl*_*faecalis*_	ATCAAGTACAGTTAGTCTTTATTAG	941	55 °C	[13]
	ACGATTCAAAGCTAACTGAATCAGT			
*ddl*_*faecium*_	TTGAGGCAGACCAGATTGACG	658	55 °C	[13]
	TATGACAGCGACTCCGATTCC			
*van A*	CATGAATAGAATAAAAGTTGCAATA	1030 bp	54 °C	[14]
	CCCCTTTAACGCTAATACGATCAA			
*van B*	GTGACAAACCGGAGGCGAGGA	433 bp	54 °C	[14]
	CCGCCATCCTCCTGCAAAAAA			
*van C*	GAAAGACAACAGGAAGACCGC	796 bp	54 °C	[15]
	ATCGCATCACAAGCACCAATC			
*aac(6′)-Ie-aph(2″)-Ia*	CAGGAATTTATCGAAAATGGTAGAAAAG	369 bp	55 °C	[16]
	CACAATCGACTAAAGAGTACCAATC			
*aph(3′)-IIIa*	GGCTAAAATGAGAATATCACCGG	523 bp	55 °C	[16]
	CTTTAAAAAATCATACAGCTCGCG			
*ant(4′)-Ia*	CAAACTGCTAAATCGGTAGAAGCC	294 bp	55 °C	[16]
	GGAAAGTTGACCAGACATTACGAACT			
*aph(2″)-Ic*	CCACAATGATAATGACTCAGTTCCC	444 bp	55 °C	[16]
	CCACAGCTTCCGATAGCAAGAG			
*aph(2″)-Ib*	CTTGGACGCTGAGATATATGAGCAC	867 bp	55 °C	[16]
	GTTTGTAGCAATTCAGAAACACCCTT			
*aph(2″)-Id*	GTGGTTTTTACAGGAATGCCATC	641 bp	55 °C	[16]
	CCCTCTTCATACCAATCCATATAACC			
*ant(3″)-III*	TGATTTGCTGGTTACGGTGAC	284 bp	55 °C	[17]
	CGCTATGTTCTCTTGCTTTTG			
*ant(6′)-Ia*	ACTGGCTTAATCAATTTGGG	596 bp	55 °C	[17]
	GCCTTTCCGCCACCTCACCG			
*PAI*	GACGCTCCCTTCTTTTGAC	387 bp	54 °C	[18]
	CCAGAGAAATTACTACCAT			
*sprE*	GGTAAACCAACCAAGTGAATC	300 bp	56 °C	[18]
	TTCTTCCGATTGACGCAAAA			
*ace*	CAGGCCAACATCAAGCAACA	125 bp	58 °C	[18]
	GCTTGCCTCGCCTTCTACAA			
*gelE*	CGAAGTTGGAAAAGGAGGC	372 bp	54 °C	[18]
	GGTGAAGAAGTTACTCTGA			
*hyl*	ACAGAAGAGCTGCAGGAAATG	276 bp	55 °C	[19]
	GACTGACGTCCAAGTTTCCAA			
*cylA*	ACTCGGGGATTGATAGGC	688 bp	56 °C	[19]
	GCTGCTAAAGCTGCGCTT			
*esp*	TTTGGGGCAACTGGAATAGT	407 bp	56 °C	[18]
	CCCAGCAAATAGTCCATCAT			

### Antimicrobial susceptibility testing

Susceptibility testing to vancomycin (30 μg), ampicillin (10 μg), tetracycline (30 μg), linezolid (30 μg), gentamicin (120 μg), chloramphenicol (30 μg), fosfomycin (200 μg), quinupristin-dalfopristin (15 μg), streptomycin (300 μg) and ciprofloxacin (5 μg) (MAST, Merseyside, U.K) was assessed according to the Clinical and Laboratory Standards Institute guidelines (CLSI) [20]. Multidrug resistance was defined as resistance to three or more different classes of antibiotics. Minimum inhibitory concentration (MIC) of vancomycin was determined using the agar dilution method according to CLSI guidelines [20]. The MIC was recorded as the lowest concentration that completely inhibited growth except for a single colony or a faint haze caused by the inoculum. *Enterococcus faecalis* ATCC29212 was used as reference strain for susceptibility testing.

### DNA extraction

Enterococcal DNA was extracted by suspending a loop of overnight colonies in a tube containing 100 μl TE buffer (10 mM Tris-HCl; 1 mM EDTA, pH 8.0) (Merck, Germany) and 0.5 μl lysozyme (100 mg/ml) (Sigma-Aldrich, USA), and incubated at 37 °C for 1 h. The suspensions boiled for 10 min and centrifuged at 14,000 rpm for 5 min at room temperature [11]. The supernatants were collected and stored at − 20 °C as DNA template stocks. The concentration and purity of DNA samples were determined using a NanoDrop Spectrophotometer (ND-1000, Nano-Drop Technologies, Wilmington, DE) at 260 and 260/280 nm, respectively.

### Detection of resistance and virulence genes

The presence of vancomycin resistance genes *vanA, vanB, vanC,* aminoglycoside resistance genes *aac (6′)-Ie-aph (2“)-Ia, aph (3’)-IIIa, ant (4’)-Ia, aph (2”)-Ic, aph (2“)-Ib, aph (2”)-Id, ant (3″)-III, ant (6′)-Ia* and virulence genes *gelE, PAI, esp, ace, cyl, hyl* and *sprE* was assessed using PCR method (The primers [Metabion, Germany] are shown in Table 1) [21–27]. Polymerase chain reaction was performed using DreamTaq PCR Master Mix (Ampliqon, Denmark), which contains Taq polymerase, dNTPs, MgCl2 and the appropriate buffer. Each PCR tube contained 25 μl reaction mixture composed of 12.5 μl of the master mix, 1.5 μl of each forward and reverse primer solution (in a final concentration of 200 nM), 5 μl of DNA with concentration of 100 ng/μl and nuclease-free water to complete the final volume. Amplification was performed using the Gene Atlas 322 system (ASTEC, Japan) with initial denaturation at 94 °C, 5 min followed by 30 cycles of denaturation (94 °C, 1 min), annealing (54–58 °C, 45 s) and extension (72 °C, 1 min), with a final extension step (72 °C, 10 min). The amplified DNA was separated by submarine gel electrophoresis, stained with ethidium bromide and visualized under UV transillumination (UVITEC, UK). *Enterococcus faecalis* ATCC 51299 and *E. faecalis* MMH594 was used as the positive control strain.

### Phenotypic detection of virulence factors

#### Cytolysin activity

Cytolysin activity was assessed on Brain Heart Infusion (BHI) Agar (Merck, Germany) supplemented with 5% horse blood. Cytolytic activity was detected after 24 h incubation at 37 °C as β-hemolysis surrounding bacterial colonies [28]. All assays were performed in triplicate.

#### Gelatinase activity

Gelatinase activity was assessed using 3% gelatin medium (Merck, Germany) as described previousely [12]. All assays were performed in triplicate.

#### Haemagglutination assay

Haemagglutination assay was performed according to Elsner et al. [12]. Enterococcal isolates were incubated for 24 h at 37 °C on BHI Agar (Merck, Germany) supplemented with 10% sheep blood. Bacterial suspension with final concentration of 1.8 × 10^9^ CFU/mL was prepared in phosphate-buffered saline (PBS). Then, 50 μ1 of bacterial suspension mixed gently with 50 μl of 3% human erythrocyte (collected from healthy volunteer people) suspension in PBS (pH 7.4) in 96-well U-bottom microtiter plates. Haemagglutination was recorded after rotating the plates for 5 min and then keeping them at room temperature for 30 min. All assays were performed in triplicate.

#### Biofilm forming assay

Biofilm forming capacity was determined using microtiter plate as described by Zeighami et al. [29]. Biofilm formation was scored as follows:**_**, non-biofilm forming (A595 < 1); +, weak (1 < A595 ≤ 2); ++, moderate (2 < A595 ≤ 3); +++, strong (A595 > 3). Reported values are the mean of three measurements.

#### Statistical analysis

The data were analyzed with SPSS version 17.0 software (SPSS, Inc., Chicago, IL). A chi-square and Fisher s Exact tests were used to determine the statistical significance of the data. A *P* value of < 0.05 was considered significant.

## Results

### Patient demographics

A total of 100 enterococci were collected from urine samples of hospitalized patients with symptomatic UTI. Among the total patients, 48 (48%) patients were younger than 30 years, 36 (36%) were 30–45 years and 16 (16%) were > 45 years. The sex distribution was 64 (64%) female and 36 (36%) male. Of 100 enterococci, 69 isolates were identified as *E. faecalis*, 10 isolates as *E. faecium* and 21 isolates as “other” *Enterococcus* species.

### Antimicrobial susceptibility

Antibiotic resistance profile of isolates is presented in Table 2. Overall, 93 isolates were resistant to one or more antimicrobial agents, with the most frequent resistance found against tetracycline (86%), ciprofloxacin (73%) and quinupristin-dalfopristin (53%). Gentamicin and streptomycin resistance was detected in 50 and 34% of isolates, respectively. Fosfomycin showed the highest activity against isolates and only one isolate was fosfomycin resistant. Furthermore, 95% of isolates were susceptible to linezolid.

**Table 2:** Antimicrobial resistance of *Enterococcus* species

Antimicrobial agents	No. (%) ofresistant*E. faecalis*(*n* = 69)	No. (%) ofresistant*E. faecium*(*n* = 10)	No. (%) ofresistant otherEnterococcalspp. (*n* = 21)	No. (%) oftotalresistantisolates(*n* = 100)
Vancomycin	0 (0)	7 (70)	14 (66.6)	21 (21)
Ampicillin	0 (0)	10 (100)	12 (57.1)	22 (22)
Tetracycline	62 (89.8)	9 (90)	15 (71.4)	86 (86)
Gentamicin	31 (45)	7 (70)	12 (57.1)	50 (50)
Linezolid	4 (5.8)	0	1 (4.76)	5 (5)
Ciprofloxacin	47 (68.1)	9 (90)	17 (80.9)	73 (73)
Chloramphenicol	16 (23.2)	2 (20)	3 (14.3)	21 (21)
Fosfomycin	1 (1.44)	0 (0)	0 (0)	1 (1)
Quinupristin-Dalfopristin	34 (34.8)	6 (60)	13 (61.9)	53 (53)
Streptomycin	19 (27.5)	5 (50)	10 (47.6)	34 (34)

Twenty one isolates were resistant to vancomycin, with MICs≥32 μg/ml, and 15% of isolates showed MIC of vancomycin ≥256 μg/ml and considered as high level vancomycin resistant (HLVR) [30]. Of 21 vancomycin resistant enterococci, 10 isolates were identified as *E. faecium* and 11 isolates as “other” *Enterococcus* species. No ampicillin or vancomycin resistant *E. faecalis* isolate was detected.

A total of 36 isolates were resistant to at least three different classes of antimicrobial agents and considered as MDR. The most prevalent MDR pattern was resistance to tetracycline, ciprofloxacin, gentamicin and quinupristin-dalfopristin.

### Distribution of vancomycin and aminoglycoside resistance genes

Among 21 VRE isolates, 12 (57.1%) isolates were positive for the presence of *van* genes. Ten *E. faecium* (47.6%) isolates carried *vanA* and 2 (9.5%) *Enterococcus* species carried *vanC.*

The distribution of aminoglycoside resistance genes (ARGs) is presented in Table 3. The most prevalent ARG was *ant (3“)-III* (78%), followed by *aph (3’)-IIIa* (67%), *ant (6’)Ia* (62%) and *aac (6’)-Ie-aph (2”)-Ia* (15%). The frequency of *aph (2″)-Ib* and *ant (4′)-Ia* was 7 and 4%, respectively. Frequency of ARGs in *E. faecium* and *E. faecalis* isolates did not show significant difference.

**Table 3:** Frequency of vancomycin and aminoglycoside resistance genes in *Enterococcus* species

Genes	No. (%) of *E. faecalis (n* = 69)	No. (%) of *E. faecium (n* = 10)	No. (%) of Other species (*n* = 21)	No. (%) of Total(*n* = 100)
*aac(6′)-Ie-aph(2″)-Ia*	10 (14.5)	1 (10)	4 (19)	15 (15)
*aph(3′)-IIIa*	46 (66.6)	7 (70)	14 (66.7)	67 (67)
*ant(4′)-Ia*	3 (4.3)	0 (0)	1 (4.7)	4 (4)
*aph(2″)-Ic*	6 (8.7)	0 (0)	2 (9.5)	8 (8)
*aph(2″)-Ib*	4 (5.8)	2 (20)	1 (4.7)	7 (7)
*aph(2″)-Id*	5 (7.2)	2 (20)	1 (4.7)	8 (8)
*ant(3″)-III*	50 (72.5)	9 (90)	19 (90.5)	78 (78)
*ant(6′)-Ia*	41 (59.4)	6 (60)	15 (71.4)	62 (62)
*vanA*	0	10 (100)	0	10 (10)
*vanB*	0	0	0	0
*vanC*	0	0	2 (9.5)	2 (2)

The presence of multiple ARGs with different combinations was found in enterococci. Eighty percent of *E. faecium* and 78.3% of *E. faecalis* isolates were carried two or more ARGs (Table 4). The number of ARGs per isolate and their specific combinations are shown in Table 4. The most frequent combinations of ARGs in enterococci were *ant (6′)Ia + ant (3′)-IIIa + aph (3′)-IIIa* (15.2%), followed by *aph (3′)-IIIa + ant (3′)-III* (12.6%) and *ant (6′)Ia + aph (3′)-IIIa* (10.1%).

**Table 4:** Frequency of different combinations of ARG among *Enterococcus species*

No. of ARG	ARG combinations	No. (%) of ARGs in *E. faecalis* (*n* = 69)	No. (%) of ARGs in *E. faecium* (*n* = 10)	Total No. (%) (*n* = 79)
Without ARG	–	1 (1.4)	–	1 (1.2)
1 ARG	*ant(6′)Ia*	5 (7.2)	–	16 (20.2)
	*ant(3′)-IIIa*	7 (10.1)	2 (20)	
	*aph(3′)-IIIa*	2 (2.8)	–	
2 ARGs	*aph(3′)-IIIa + ant(3′)-III*	8 (11.5)	2 (20)	26 (32.9)
	*ant(6′)Ia + ant(3′)-IIIa*	4 (5.7)	–	
	*ant(6′)Ia + aph(3′)-IIIa*	8 (11.5)	–	
	*ant(3′)-IIIa + aac (6′)-Ie*	1 (1.4)	–	
	*ant(3′)-IIIa + aph(3′)-IIIa*	2 (2.8)	–	
	*ant(6′)Ia + aac (6′)-Ie*	1 (1.4)	–	
3 ARGs	*ant(3′)-IIIa + aph(2″)-Ic + ant(4′)Ia*	1 (1.4)	–	22 (27.8)
	*ant(6′)Ia + ant(3′)-IIIa + aph(3′)-IIIa*	10 (14.4)	2 (20)	
	*ant(6′)Ia + aph(3′)-IIIa + aph(2″)-Ic*	1 (1.4)	–	
	*ant(3′)-IIIa + aph(3′)-IIIa + ant(4′)Ia*	1 (1.4)	–	
	*ant(3′)-IIIa + aph(3′)-IIIa + aph(2″)-Ic*	1 (1.4)	–	
	*ant(3′)-IIIa + aph(3′)-IIIa + aac (6′)-Ie*	1 (1.4)	–	
	*ant(6′)Ia + aac (6′)-Ie + aph(2″)-Ic*	1 (1.4)	–	
	*ant(6′)Ia + ant(3′)-IIIa + aph(2″)-lb*	1 (1.4)	1 (10)	
	*ant(6′)Ia + aph(3′)-IIIa + aac (6′)-Ie*	–	1 (10)	
	*ant(3′)-IIIa + aph(2″)-ld + aph(3′)-IIIa*	1 (1.4)	–	
4 ARGs	*ant(6′)Ia + ant(3′)-IIIa + aph(2″)-ld + aph(3′)-IIIa*	2 (2.8)	1 (10)	12 (15.1)
	*ant(6′)Ia + ant(3′)-IIIa + aph(3′)-IIIa + aac (6′)-Ie*	4 (5.7)	–	
	*ant(6′)Ia + ant(3′)-IIIa + aph(2″)-lb + aph(3′)-IIIa*	1 (1.4)	–	
	*ant(3′)-IIIa + aph(2″)-ld + aph(3′)-IIIa + aph(2″)-Ic*	1 (1.4)	–	
	*ant(6′)Ia + ant(3′)-IIIa + aph(2″)-ld + aac (6′)-Ie*	1 (1.4)	–	
	*ant(6′)Ia + ant(3′)-IIIa + aph(3′)-IIIa + aph(2″)-Ic*	1 (1.4)	–	
	*ant(3′)-IIIa + aph(2″)-lb + aph(3′)-IIIa + ant(4′)Ia*	1 (1.4)	–	
5 ARGs	*ant(6′)Ia + ant(3′)-IIIa + aph(2″)-lb + aph(3′)-IIIa + aac (6′)-Ie*	1 (1.4)	–	2 (2.5)
	*ant(6′)Ia + ant(3′)-IIIa + aph(2″)-lb + aph(2″)-ld + aph(3′)-IIIa*	**–**	1 (10)	

### Distribution of enterococcal virulence related genes

The virulence related genes *PAI, sprE, ace, gelE, hyl, cylA* and *espE* were detected in *E. faecalis* and *E. faecium* isolates*.* The frequency of enterococcal virulence genes is shown in Table 5. The most frequent virulence gene was *ace* (88.6%), followed by *esp* (67.1%), *PAI* (45.5%) and *sprE* (41.7%). As shown in Table 5, the frequency of *ace, cylA* and *esp* genes among *E. faecalis* isolates was significantly higher than *E. faecium* (*P* < 0.05). All *E. faecalis* isolates carried at least one virulence gene. However, *gelE*, and *cylA* genes were not detected in *E. faecium* isolates.

**Table 5:** Frequency of virulence genes among *Enterococcus species*

virulencegenes	*E. faecalis*(*n* = 69)	*E. faecium*(*n* = 10)	*P* value	Total(*n* = 79)
*PAI*	31 (44.9)	5 (50)	0.488	36 (45.5)
*sprE*	30 (43.4)	3 (30)	0.142	33 (41.7)
*ace*	62 (89.8)	8 (80)	0.001*	70 (88.6)
*gelE*	17 (24.6)	0	0.063	17 (21.5)
*hyl*	2 (2.8)	1 (10)	0.337	3 (3.8)
*cylA*	25 (36.2)	0	0.017*	25 (31.6)
*esp*	50 (72.4)	3 (30)	0.012*	53 (67.1)

Fisher s Exact test was used to determine the statistical significance of the data

*P value of < 0.05 was considered significant

Several different combinations of virulence genes were found in enterococci. Table 6 shows that 97.1% of *E. faecalis* and 50% of *E. faecium* isolates harbored two or more virulence genes simultaneously (*P* < 0.05). The mean number of virulence genes per isolate was higher in *E. faecalis* isolates than *E. faecium* (*P* < 0.05). The most frequent combination in *E. faecalis* was *PAI-sprE-ace-esp* (13%), followed by*PAI-ace-cylA-esp* and *ace-cylA-esp* (8.6%).

**Table 6:** Frequency of different combinations of virulence genes among *Enterococcus species*

Genetic profile	No. (%) of *E. faecalis*(*n* = 69)	No. (%) of *E. faecium*(*n* = 10)
No virulence factor	–	–
*PAI*	–	1 (10)
*Ace*	2 (2.8)	4 (40)
*PAI-ace*	1 (1.4)	–
*sprE-ace*	2 (2.8)	–
*ace-gelE*	1 (1.4)	–
*ace-hyl*	1 (1.4)	1 (10)
*PAI-esp*	1 (1.4)	1 (10)
*ace-esp*	4 (5.7)	–
*gelE-esp*	2 (2.8)	–
*cylA-esp*	1 (1.4)	–
*PAI-sprE-ace*	4 (5.7)	1 (10)
*sprE-ace-gelE*	3 (4.3)	–
*PAI-ace-cylA*	1 (1.4)	–
*ace-gelE-cylA*	1 (1.4)	–
*ace-hyl-cylA*	1 (1.4)	–
*PAI-sprE-esp*	2 (2.8)	–
*PAI-ace-esp*	5 (7.2)	–
*sprE-ace-esp*	4 (5.7)	–
*ace-gelE-esp*	2 (2.8)	–
*ace-cylA-esp*	6 (8.6)	–
*sprE-ace-gelE-cylA*	2 (2.8)	–
*PAI-sprE-ace-esp*	9 (13)	2 (20)
*sprE-ace-gelE-esp*	1 (1.4)	–
*PAI-ace-cylA-esp*	6 (8.6)	–
*sprE-gelE-cylA-esp*	1 (1.4)	–
*ace-gelE-cylA-esp*	4 (5.7)	–
*PAI-sprE-ace-cylA-esp*	2 (2.8)	–

### Phenotypic detection of virulence factors

Gelatinase and cytolytic activity, haemagglutination and biofilm formation was assayed by phenotypic tests (Table 7). Hemolytic activity of *E. faecalis* isolates (46.4%) was higher than other *Enterococcus* spp. (14.3%). Hemolytic activity cannot be detected in *E. faecium* isolates. Gelatinase activity was detected in 25% of enterococci. However, there was no significant difference among *Enterococcus* species in gelatinase activity (*P* > 0.05). Haemagglutination and biofilm formation phenotypes were detected in 75 and 74% of enterococci. Biofilm formation in *E. faecalis* isolates (89.8%) was significantly higher than other species. Of 74 biofilm forming isolates, 64 isolates produced weak biofilm (+) and 10 isolates formed moderate biofilm (++). Frequency of resistance genes among virulence factors producing entrococci is shown in Table 8. The resistance genes of *aph (3′)-IIIa*, *ant (3′)-III* and *ant (6′)Ia* were detected significantly higher among enterococci with haemagglutination and biofilm formation phenotypes (*P* < 0.05). Also, all resistance genes (except *vanC, ant (4′)Ia* and *aph (3′)-IIIa*) were frequently detected in isolates with gelatinase and cytolytic activity (P < 0.05).

**Table 7:** Phenotypic detection of virulence factors in *Enterococcus species*

Virulencefactors	*E. faecalis* (*n* = 69) No. (%)	*E. faecium* (*n* = 10) No. (%)	Other *spp*.(*n* = 21)No. (%)	*P* value	Total(*n* = 100)No. (%)
Cytolytic activity	32 (46.4)	0	3 (14.3)	0.001*	35 (35)
Gelatinase activity	18 (26.1)	0	7 (33.3)	0.107	25 (25)
Hemagglutination	55 (79.7)	7 (70)	13 (61.9)	0.245	75 (75)
Biofilm formation	62 (89.8)	2 (20)	10 (47.6)	0.000*	74 (74)

Fisher s Exact test was used to determine the statistical significance of the data

*P value of < 0.05 was considered significant

**Table 8:** Frequency of resistance genes among virulence factors producing entrococci

Resistance genes	Van A(*n* = 10)	Van C (*n* = 2)	*aac(6′)-Ie-aph(2″)-Ia (n = 20)*	*aph(3′)-IIIa (n = 71)*	*ant(4′)-Ia (n = 6)*	*aph(2″)-Ic (n = 9)*	*aph(2″)-Ib (n = 5)*	*aph(2″)-Id (n = 7)*	*ant(3″)-III (n = 84)*	*ant(6′)-Ia (n = 57)*
Virulence factors										
Cytolytic activity (*n* = 35)	8*	1	14*	20*	4	8*	4*	6*	34*	32*
Gelatinase activity (*n* = 25)	8*	2	18*	16	6*	9*	4*	7*	25*	25*
Hemagglutination (*n* = 75)	7	1	13	60*	5	9	5	7	74*	53*
Biofilm formation (*n* = 74)	10	1	17	66*	6	8	5	7	74*	56*

Fisher s Exact test was used to determine the statistical significance of the data

*P value of < 0.05 was considered significant

## Discussion

Multidrug resistant enterococci, as important nosocomial pathogens, have become a serious problem in hospitalized patients [3, 6]. Due to the extensive misuse of antimicrobial agents in our country, treatment of infections associated with MDR enterococci is complicated [31, 32]. In our study, 93% of enterococci were resistant to one or more antimicrobial agents and 36% were MDR. The frequency of antimicrobial resistance among *E. faecium* isolates was more than *E. faecalis* (except for linezolid and fosfomycin). The inherent antibiotic resistance and dissemination of resistance genes through conjugative transposons and plasmids play an important role in development of MDR enterococci [33]. High frequency of antimicrobial resistance among enterococci was reported in previous studies from Iran [3, 32, 34].

While intrinsic mechanisms result in low level aminoglycoside resistance, acquisition of mobile genetic elements typically underlies high level aminoglycoside resistance in *E. faecium* and *E. faecalis* [4]. High level aminoglycoside resistance among enterococci was first reported in France in 1979 and since then has caused serious problems in healthcare settings worldwide [6]. Recent studies indicated that HLGR among enterococci to be more common than HLSR [35]. Similarly, gentamycin resistant enterococci (50%) were detected with higher frequency than streptomycin resistance (34%) in our study. Also, Mirnejad et al. and Zarrilli et al. were reported that 56.9 and 46.1% of enterococci were HLGR, respectively [9, 13].

Aminoglycoside resistance genes in *E. faecium* isolates were detected with higher frequency than *E. faecalis,* an observation which is consistent with that found in previous reports [6, 35]. While previous studies found that *aac (6′)-Ie-aph (2″)-Ia* was the most common ARG [6, 9, 35], we detected *aac (6′)-Ie-aph (2″)-Ia* with lower frequency in 15% of enterococci. According to our results, the most prevalent ARG was *ant (3″)-III* (78%), followed by *aph (3′)-IIIa* (67%), *ant (6′)Ia* (62%).

Furthermore, 80 % of *E. faecium* and 78.3% of *E. faecalis* isolates were carried two or more ARGs. Our results are consistent with previous reports on the predominance of enterococci with two or more ARGs [9, 35] .

High level vancomycin resistance, leading causes of hospital-acquired infections, were first reported in United Kingdom in 1980s and since then have caused significant public health concern because of its propensity to acquire and transfer the mobile resistance genes [14]. As reported in previous studies, the most common risk factors for VRE infections are prolonged hospitalization, use of vancomycin and third-generation cephalosporins and chronic dialysis [15]. In our study, 15% of enterococci were high level vancomycin-resistant with MIC of ≥256 μg/ml and 21% were vancomycin resistant which consistent with some previous reports [15, 16]. No vancomycin resistant *E. faecalis* (VRE_*fs*_) was found in our study. In contrast to our results, the frequency of VRE_*fs*_ was higher than vancomycin resistant *E. faecium* (VRE_*fm*_) in study carried out by Sabouni et al. in Iran [16]. Similar to our study, Wisplinghoff et al. reported vancomycin resistance in 2% of *E. faecalis* and 60% of *E. faecium* isolates. However, they did not report high level vancomycin resistance [17]. National survey data have indicated the prevalence of VRE in 0–59% of isolates in 126 adult ICUs from 60 US hospitals [18]. Several reports also showed the elevated occurrence of *vanA* in comparison to other *van* types [5, 16, 17]. We found a high occurrence of *vanA* in VRE isolates. Several hospitals located in São Paulo and other Brazilian cities reported both outbreaks and isolated cases of VRE infection/colonization [19]. All VRE_*fm*_ isolates carried *vanA* but *vanB* was not detected among enterococci in our study. Similar to our results, Cekin et al. did not detected *vanB* among enterococci [31].

The essential virulence factors for pathogenicity of enterococci have not yet been described and the pathogenicity has been considered a multifactorial process [10]. Previous studies showed the association between the presence of virulence factors and promoting emergence of enterococcal infections in nosocomial settings [5, 10]. Our results showed different prevalence of virulence genes in enterococci which ranged from 3.9 to 8.6%. The most frequent virulence genes were *ace* and *esp*. Ace is an adhesion of collagen from *Enterococcus* that binds to collagen and laminin and belongs to the MSCRAMM family. In Bulgaria, Strateva et al. reported varied distribution of *esp* in non-invasive *E. faecalis* isolates (54.3–64.8%) compared to invasive isolates (33.3%) [2]. In our study, *ace* and *esp* were found respectively among 88.6 and 67.1% of enterococci isolated from UTIs, which confirm the important role of Ace and Esp as colonization factors in UTIs. The frequency of *ace* and *esp* in *E. faecalis* isolates was significantly higher than *E. faecium.* A strong correlation between the presence of Esp and the ability of an *Enterococcus* isolate to colonizes and persists in urinary tract and forms biofilm in vitro has been reported [10]. According to our results, 74% of enterococci showed biofilm formation phenotype which exhibits an important role of biofilm formation in UTIs. The virulence genes *gelE* and *cylA* were not detected in *E. faecium* isolates. Similar to our results, a multicenter study on distribution of virulence determinants in fecal *E. faecium* isolates of patients in 13 hospitals from nine European countries showed total absence of *gelE* gene. However, 26 and 36.2% of *E. faecalis* isolates carried *gelE* and *cylA* determinants, respectively. The least prevalence among enterococci was *hyl* which was detected in only 3 isolates (3.9%). Similar to our results, Soheili et al. demonstrated that only 8% of *E. faecalis* isolates in Malaysian patients carried *hyl* [1]. Since *hyl* was not prevalent in our study and some previous reports [1, 28], we believe that this gene could has little role in pathogenicity of *Enterococcus* in comparison with other prevalent virulence genes.

According to our results, 97.1% of *E. faecalis* and 50% of *E. faecium* isolates harbored two or more virulence genes simultaneously (*P* < 0.05). Furthermore, among gentamycin resistant enterococci, 45 isolates (90%) were carried at least two or more virulence genes which is consistent with previous reports [2, 6, 35]. Also, 42% of VRE isolates were harbored at least two or more virulence determinants.

One of the limitations of our study was the low number of *E. faecium* isolates and since the study was conducted in small geographical area, Northwest of Iran, the results cannot be generalized. For better characterization of enterococci strains, we suggest that molecular typing methods such as pulsed-field gel electrophoresis and multilocus sequence typing will be done.

## Conclusion

Our study demonstrated that *E. faecalis* was more common than other *Enterococcus* species, but high frequency of aminoglycoside and vancomycin resistance was detected among *E. faecium* isolates*.* The distribution of virulence genes (except *hyl*) among *E. faecalis* isolates was higher than *E. faecium*. Due to high frequency of MDR enterococci, it seems that the appropriate surveillance and control measures are essential to prevent the emergence and transmission of these isolates in hospitals. Further studies should be carried out for a better understanding of the association between the presence of virulence determinants and emergence of multidrug resistant enterococci.

## Data Availability

The datasets will not be available on a publically available website, but it may be possible to provide access to anonymized data. Anyone who wants to request the data can contact with Habib Zeighami, corresponding author.

## Abbreviations

AAC: Aminoglycoside acetyl transferase
AME: Aminoglycoside modifying enzyme
ANT: Aminoglycoside nucleotidyl transferase
APH: Aminoglycoside phosphoryl transferase
ARG: Aminoglycoside resistance gene;
HLAR: High level aminoglycoside resistance
MDR: Multidrug resistant
PYR: L-Pyrrolidonyl-β-Naphthylamide
UTI: Urinary tract infection
VRE: Vancomycin resistant enterococci
